# Affinity-tuned mesothelin CAR T cells demonstrate enhanced targeting specificity and reduced off-tumor toxicity

**DOI:** 10.1172/jci.insight.186268

**Published:** 2024-11-22

**Authors:** Yanping Yang, Yogindra Vedvyas, Yago Alcaina, Sydney J. Trumper, Diella S. Babu, Irene M. Min, Jacqueline M. Tremblay, Charles B. Shoemaker, Moonsoo M. Jin

**Affiliations:** 1 Department of Radiology, Houston Methodist Research Institute, Houston, Texas, USA.; 2 Department of Radiology, Weill Cornell Medicine, New York, New York, USA.; 3 Department of Infectious Disease and Global Health, Cummings School of Veterinary Medicine, Tufts University, North Grafton, Massachusetts, USA.

**Keywords:** Oncology, Therapeutics, Cancer immunotherapy, Cellular immune response, Mouse models

## Abstract

The application of chimeric antigen receptor (CAR) T cell therapy in solid tumors is hindered by life-threatening toxicities resulting from on-target, off-tumor killing of nonmalignant cells that express low levels of the target antigen. Mesothelin (MSLN) has been identified as a target antigen for CAR T cell treatment of mesothelioma, lung, ovarian, and other cancers because of its high expression on tumor cells and limited expression on mesothelial cells. However, fatal off-tumor toxicity of high-affinity MSLN-targeting CAR T cells has been reported in multiple clinical trials. In this study, we constructed CARs using mutant variants of a single-domain nanobody that bind both human and mouse MSLN with a wide range of affinities and examined tumor responses and their toxicities from on-target, off-tumor interactions in mouse models. CAR T cells with low nanomolar affinity (equilibrium dissociation constant, *K_D_*) exhibited profound systemic expansion with no apparent infiltration into the tumor. With a gradual reduction of CAR affinity toward the micromolar *K_D_*, the expansion of CAR T cells became more restricted to tumors. Our preclinical studies demonstrated that high-affinity MSLN CARs were associated with fatal on-target, off-tumor toxicity and that affinity-tuned CARs rendered T cells more selective for MSLN-high tumors.

## Abstract 2

Affinity-tuned chimeric antigen receptor (CAR) T cells selectively target antigen-high tumors, reducing off-tumor toxicity and improving safety and efficacy in treating solid tumors.

## Introduction

Mesothelin (MSLN) is a glycophosphatidyl inositol–anchored surface glycoprotein and is physiologically expressed on mesothelial cells lining the pleura, peritoneum, and pericardium (1). Its overexpression in a wide range of solid cancers, combined with its low expression on normal mesothelial cells, makes MSLN an attractive tumor antigen for targeted cancer therapy, including T cell–based therapies (2). Additionally, aberrant expression of MSLN has been associated with tumor aggressiveness, malignant transformation, increased invasion and metastasis, and resistance to apoptosis (3–6). These characteristics underscore the potential utility of MSLN-targeting therapies to eradicate aggressive cancer cells and reduce the likelihood of tumor relapse.

Chimeric antigen receptor (CAR) T cell therapy has achieved considerable clinical success in patients with hematologic malignancies (7). However, delivering meaningful clinical activity in solid cancers by the CAR T cell therapy, including CAR T cells targeting MSLN, remains challenging (8). This could be due to factors such as inadequate CAR T cell infiltration into the tumor, inhibition of CAR T cell function by the immunosuppressive tumor microenvironment, and the risk of on-target, off-tumor toxicity resulting from CAR T cell recognition of healthy cells expressing the target antigen. CAR molecules typically incorporate high-affinity single-chain variable fragments (scFvs) in their binding domains to maximize tumor killing. However, the off-tumor targeting of high-affinity CARs is particularly concerning because of CAR T cells’ high sensitivity in recognizing low-level antigen expression, which can be greater than that of monoclonal antibodies (9). Severe pulmonary toxicity and patient fatalities have been reported in clinical trials evaluating an MSLN CAR incorporating a nanomolar affinity scFv (Clone M5, *K_D_* 27 nM) (10, 11). Another trial evaluating a TCR fusion construct containing the variable heavy chain of a single-domain anti-MSLN antibody MH1 showed mixed activity but also encountered dose-limiting toxicity (12). As a result, there remains a critical need for engineering safer and more effective MSLN CAR T cells and developing strategies to assess their safety in preclinical studies prior to clinical trials.

We have demonstrated the feasibility of tuning CAR affinity to selectively target tumors overexpressing target antigens while sparing nonmalignant cells with basal levels of antigen expression (13, 14). In the current study, we selected 1 MSLN-specific variable heavy domain of heavy chain–only antibody (VHH, also called nanobody) to construct the CAR and performed systematic affinity tuning through mutagenesis of the residues in the complementarity-determining regions (CDRs) of the VHH. Naturally, scFvs derived from antibodies selected from mouse immune libraries after immunization with human antigens are specific to human antigens. CAR molecules built with such scFvs typically do not manifest on-target, off-tumor toxicity against normal mouse tissues. In contrast, camelids can be immunized with both human and mouse antigens, enabling the discovery of antibodies that can cross-react with antigens from both species. This is particularly likely when there is a high degree of homology between human and mouse antigens. Utilizing a VHH capable of binding both human and mouse MSLN with comparable affinities as the CAR binding domain offers a distinct advantage. It allows for the assessment of on-target, off-tumor effects in mouse models in addition to on-target activity against human tumor xenografts. Through a side-by-side comparison of MSLN-specific VHH CARs with affinities spanning from subnanomolar to micromolar *K_D_*, we examined the risk of fatal on-target, off-tumor toxicity associated with high-affinity MSLN CARs. Furthermore, we demonstrated that reducing the affinity of CAR to the micromolar range can improve the tumor-targeting efficiency and safety of MSLN CAR T cells.

## Results

### Discovery of VHH that binds both human and mouse MSLN.

We immunized alpacas with C-terminal domains of recombinant human and mouse MSLN proteins and generated a VHH display phage library following methods described previously (15). We then panned the library and screened colonies for binding to human and mouse MSLN. Most clones demonstrated binding for human MSLN, while about 10% also recognized mouse MSLN. All selected clones displaying cross-reactivity to human and mouse MSLN belonged to the same clonal family, deriving from a common B cell progenitor based on CDR3 homologies. The clone that produced the strongest binding was named JZQ-B4. Nine additional clonal families were identified and found to be specific only to human MSLN. One member from each of the 10 clonal families was expressed, purified, and characterized for affinities to human and mouse MSLN by quantitative dilution ELISA. All selected clones displayed an EC_50_ value indicative of subnanomolar affinities for human MSLN, while only JZQ-B4 exhibited high affinity for mouse MSLN (Supplemental Table 1; supplemental material available online with this article; https://doi.org/10.1172/jci.insight.186268DS1).

### Single–amino acid alanine scanning using a yeast surface display system.

**Figure 1:**
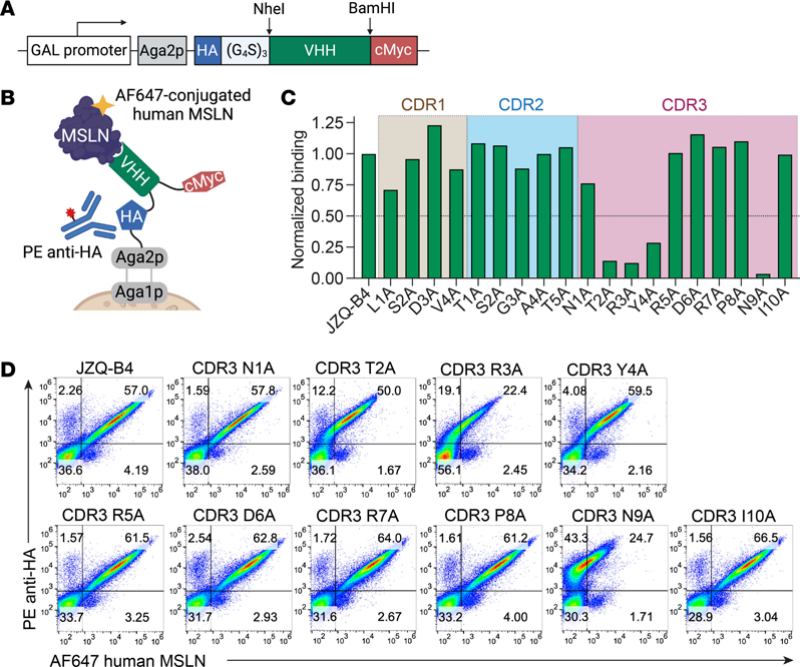
Alanine scanning of JZQ-B4 VHH using a yeast surface display system. (**A**) The schematic of the cell surface display of the VHH variants (created with BioRender.com). Different VHH variants were subcloned into the vector within NheI-BamHI restriction sites. (**B**) The schematic illustrating the topology of the fusion proteins on the yeast cell surface (created with BioRender.com). Aga2p is disulfide bonded to Aga1p, forming α-agglutinin. The HA tag at the N-terminus was used for the detection of displayed fusion proteins while Alexa Fluor 647–labeled human MSLN was used to measure the affinity of VHHs. (**C**) The bar graph shows relative binding affinities of VHH variants with alanine substitutions in CDRs, normalized to the level of the parental JZQ-B4 (*n* = 1 culture per variant). (**D**) Dot plots are shown for yeast cells expressing the parental JZQ-B4 and CDR3 VHH variants after staining with anti-HA antibody and Alexa Fluor 647–labeled human MSLN.

To develop CARs with optimal affinity to selectively target tumors overexpressing MSLN while sparing healthy tissues, we generated a yeast surface display system for affinity tuning of JZQ-B4 VHH. This system allows the display of fusion proteins on cell surface comprising the Aga2p, hemagglutinin (HA) tag, VHH, and cMyc tag (Figure 1, A and B). The surface expression of the fusion proteins was detected by HA tag antibody, and the affinity of VHHs was estimated by immunostaining with Alexa Fluor 647–conjugated human MSLN monomer. We performed alanine substitution for each of the residues in the CDRs of JZQ-B4 VHH and compared the binding affinities of different VHH variants (Figure 1C). The binding of VHHs to human MSLN was normalized by the MFIs of HA tag staining to account for variations in surface expression of the fusion proteins. Alanine substitutions in the CDR1 and CDR2 of JZQ-B4 VHH did not result in dramatic reductions in the level of binding (Figure 1C). However, in the CDR3, which is typically the major paratope in VHHs, we identified 4 VHH variants (T2A, R3A, Y4A, and N9A) that showed more than 50% reduction in MSLN binding compared with the parental JZQ-B4 VHH (Figure 1, C and D).

### Alanine substitution results in a VHH CAR with 500-fold lower affinity for MSLN.

**Figure 2:**
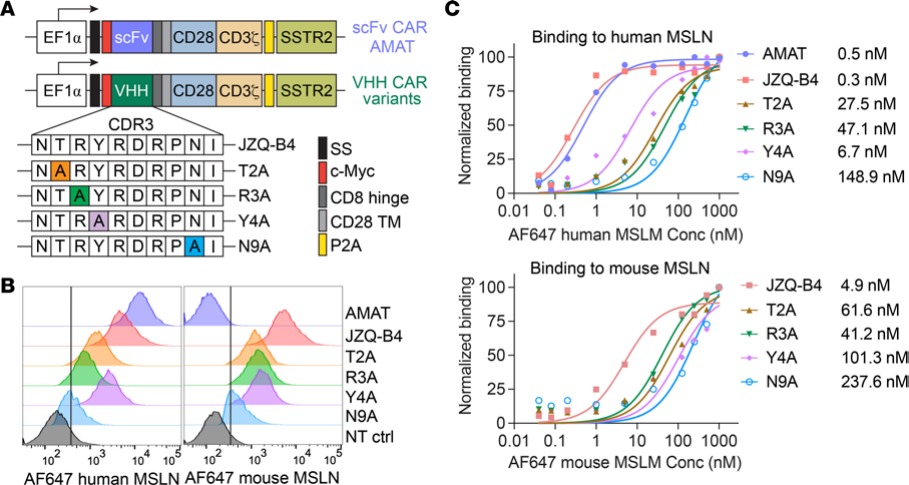
Binding affinities of MSLN CAR variants. (**A**) Schematic representation of the lentiviral vectors containing MSLN CAR variants. (**B**) Relative binding of MSLN CAR variants was estimated by staining CAR-expressing Jurkat T cells with 25 nM of Alexa Fluor 647–conjugated monomeric human and mouse MSLN, using NT cells as control. (**C**) Determination of the equilibrium binding affinities of CAR variants using a saturation binding assay with Alexa Fluor 647–conjugated human and mouse MSLN (*n* = 1).

Subsequently, we converted the JZQ-B4 VHH and the 4 variants with reduced binding (T2A, R3A, Y4A, and N9A) into CAR constructs containing CD28 and CD3ζ signaling domains (Figure 2A). All CAR constructs were designed to coexpress human somatostatin receptor 2 (SSTR2) as a genetic marker for CAR T cell imaging using positron emission tomography-computed tomography (PET/CT) (16). Additionally, we constructed an scFv-based CAR using an scFv derived from the MSLN-specific amatuximab (17), termed AMAT CAR (Figure 2A). While AMAT CAR exhibited subnanomolar affinity to human MSLN, comparable to the JZQ-B4 VHH CAR, it did not bind mouse MSLN (Figure 2, B and C). In contrast, JZQ-B4 VHH CAR demonstrated nanomolar binding affinity to mouse MSLN. Consistent with yeast surface display binding data (Figure 1, C and D), Jurkat T cells expressing T2A, R3A, Y4A, and N9A VHH CARs showed reduced binding toward MSLN compared with parental JZQ-B4 CAR (Figure 2, B and C). Among these variants, N9A CAR showed more than 100 nM *K_D_* toward human MSLN, representing an approximately 500-fold decrease in affinity compared with the parental JZQ-B4 CAR. Additionally, all VHH CAR variants with alanine substitutions maintained cross-reactivity with mouse MSLN, allowing the assessment of on-target, off-tumor toxicity in mouse models.

### Affinity-tuned N9A CAR T cells exhibit antigen density-dependent target cell lysis.

**Figure 3:**
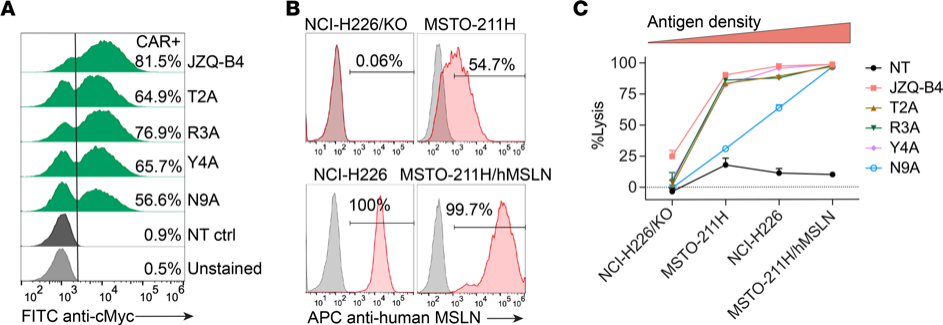
Activity of CAR T cells expressing VHH variants. (**A**) Flow cytometric histograms showing the cell surface expression of JZQ-B4 VHH CAR variants in primary CAR T cells. (**B**) Surface expression of human MSLN in tumor cell lines. NCI-H226/KO represents NCI-H226 with human MSLN knockout using a CRISPR/Cas9 system. MSTO-211H/hMSLN is MSTO-211H cell line that was transduced for human MSLN overexpression. (**C**) Bioluminescence-based cytotoxicity assay using NT cells as control. CAR T cell samples were normalized to contain the same percentage of CAR-expressing cells (50% CAR^+^) using donor-matched NT cells. Target cells were cocultured with T cells at an effector-to-target ratio of 2.5:1, and the percentages of target cell lysis were normalized to target cell–only group at 24 hours after coculture. Data represent mean ± SD of 4 technical replicates from 1 independent experiment.

We next produced primary human CAR T cells via lentiviral transduction and determined the in vitro cytotoxic activity of these VHH variant CAR T cells. In the CAR T cell products, CAR expression was determined to be between 55% and 85% by flow cytometry (Figure 3A). We compared the effector functions and cytotoxicity of these CAR T cell variants against a panel of tumor cell lines with varying levels of human MSLN expression: NCI-H226/KO (MSLN-negative), MSTO-211H (MSLN-low), NCI-H226 (MSLN-intermediate), and MSTO-211H/hMSLN (MSLN-high), which is MSTO-211H engineered to overexpress human MSLN (Figure 3, B and C). Notably, only the low-affinity N9A CAR T cells displayed antigen density-dependent target cell lysis, demonstrating increased cytotoxicity against target cells expressing higher levels of MSLN on their surface. Furthermore, the cytotoxicity of N9A CAR T cells against MSLN-high MSTO-211H/hMSLN cells was comparable to that of high-affinity JZQ-B4 CAR T cells. On the other hand, JZQ-B4 and other VHH CAR variants (T2A, R3A, and Y4A) with less than 100 nM affinity exhibited no discrimination of target cells with low, intermediate, and high levels of human MSLN expression. These high-affinity variants mediated complete lysis (close to 100%) of MSTO-211H and NCI-H226 cells expressing low and intermediate levels of MSLN within 24 hours.

### N9A CAR T cells demonstrate improved targeting specificity and tumor response in vivo.

**Figure 4:**
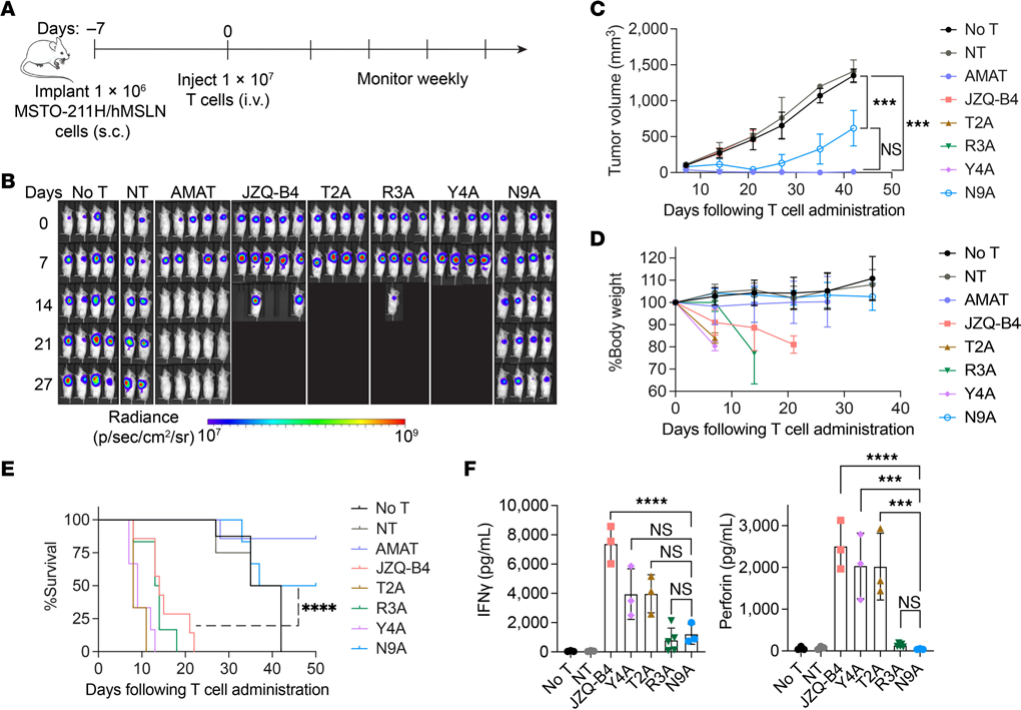
Affinity-tuned N9A CAR T cells mediate superior tumor response in vivo with reduced toxicity. (**A**) Schematic of the MSTO-211H/hMSLN tumor model. NOD.Cg-Prkdc^scid^ Il2rg^tm1Wjl^/SzJ mice were inoculated subcutaneously with 1 × 10^6^ MSTO-211H/hMSLN cells and treated with 1 × 10^7^ T cells 7 days after tumor inoculation or left untreated. (**B**) Tumor growth was monitored by whole-body bioluminescence imaging at indicated days after treatment. (**C**) Tumor size measurements. Statistical significance was determined by 2-way ANOVA with Tukey’s multiple comparisons test. (**D**) Average body weight changes relative to baseline. Data in panels **C** and **D** represent the mean ± SD from 4–8 animals per group across 2 independent experiments (2 T cell donors). (**E**) Kaplan-Meier survival curves (*n* = 4–8 mice). Significance was determined by log-rank (Mantel-Cox) test. (**F**) Cytokine levels in mouse plasma were measured on day 6 after T cell injection. Data represent the mean ± SD of 3–5 mice analyzed in 1 independent experiment. Significance was determined by 1-way ANOVA with multiple comparisons. ***P < 0.001; ****P < 0.0001.

Leveraging the cross-reactivity of JZQ-B4 variants to mouse MSLN (Figure 2B), we assessed the efficacy and toxicity of CAR affinity variants utilizing a subcutaneous mesothelioma tumor model with MSTO-211H/hMSLN cells (Figure 4A). We included AMAT scFv CAR, which lacks the cross-reactivity with mouse MSLN, to assess antitumor activity without eliciting potential on-target, off-tumor activity. As expected, AMAT CAR T cells mediated rapid tumor elimination without inducing overt toxicity and led to extended survival (Figure 4, B–E). Conversely, VHH CAR T cells (JZQ-B4, Y4A, T2A, and R3A), which possess below 100 nM affinity to both human and mouse MSLN, failed to control tumor growth and rather induced severe toxicity, resulting in substantial body weight loss and necessitating euthanasia within 3 weeks following T cell administration (Figure 4, B–E). In contrast, administration of low-affinity N9A CAR T cells resulted in tumor regression and improved survival compared with higher affinity VHH CAR T cells (Figure 4, B–E). Given that subcutaneous tumors in the control cohorts of No T (no T cell treatment) and NT (treated with nontransduced T cells) grew relatively slowly and did not cause severe toxicity, the survival benefit of N9A CAR T cells over the control cohorts was not significantly apparent in the survival analysis (Figure 4E). We followed the survival of animals only up to 50 days posttreatment, as at a later time point, animals treated with human T cells gradually and sporadically succumb to toxicity due to xenogenicity (18). Therefore, the survival data are intended for comparison among the cohorts treated with CAR affinity variants (Figure 4E), and the therapeutic benefit of CAR T cells over the control needs to be interpreted based on the difference in tumor size (Figure 4C).

Analysis of plasma samples collected on day 6 after T cell administration indicated substantially lower levels of cytokines such as IFN-γ and perforin in mice treated with N9A CAR T cells compared with those treated with high-affinity JZQ-B4, Y4A, and T2A CAR T cells (Figure 4F). We also noted much lower levels of IFN-γ and perforin in mice treated with R3A, though these animals uniformly exhibited severe toxicities as others in the parental (JZQ-B4) and other high-affinity variants (Y4A and T2A) cohorts. This is likely due to the delayed onset of severe toxicity in R3A cohort, as evidenced by the stable body weight observed on day 7, followed by a dramatic body weight loss at a later time point (Figure 4D).

**Figure 5:**
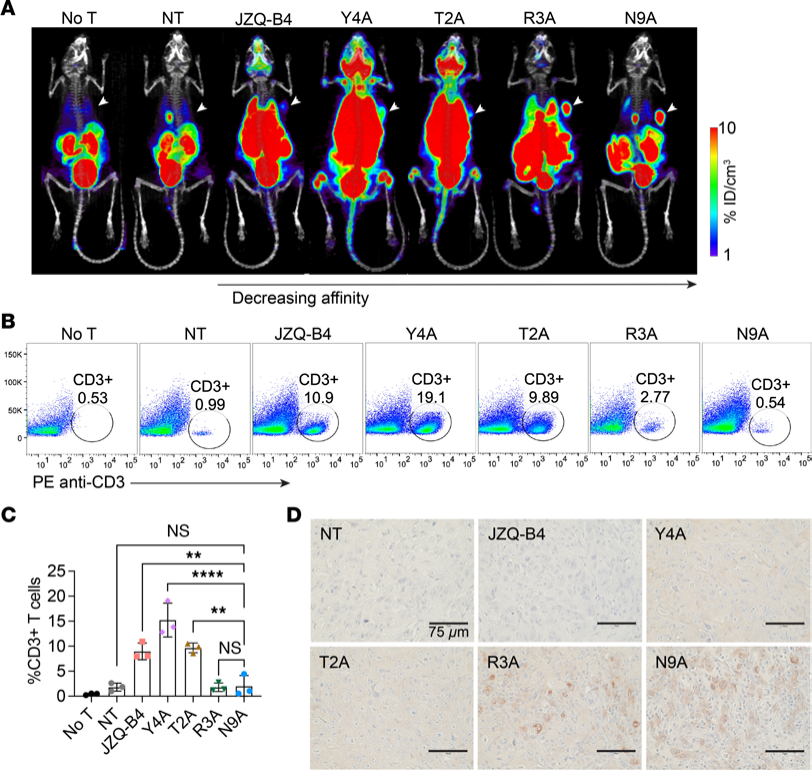
Affinity-tuned N9A CAR T cells exhibit enhanced tumor-targeting specificity with minimal off-tumor targeting in the lungs. (**A**) PET/CT images acquired on day 7 after T cell administration show tumor localization of R3A and N9A CAR T cells. Mice treated with JZQ-B4, Y4A, and T2A CAR T cells exhibited substantial CAR T cell expansion in the lungs, liver, and head. Subcutaneous tumors are indicated by white arrowheads. (**B**) On day 8 after T cell administration, mice were euthanized, and the accumulation of CD3^+^ T cells in the lungs was analyzed by flow cytometry. (**C**) The percentage of CD3^+^ T cells in the lungs (*n* = 3 mice per group from 1 independent experiment). Significance was determined by 1-way ANOVA with multiple comparisons. **P < 0.01; ****P < 0.0001. (**D**) IHC images of CD3 staining on formalin-fixed, paraffin-embedded tumor sections (40× original magnification; scale bar, 75 μm). Tumor samples were obtained on day 8 following T cell administration.

To determine if the observed toxicity of high-affinity MSLN VHH CAR T cells was due to off-tumor targeting and excessive expansion of T cells, we employed PET/CT imaging using the SSTR2-specific tracer, 1,4,7-Triazaclononane-1,4,7-triacetic acid-octreotide (^18^F-NOTA-Octreotide), to detect CAR T cell biodistribution at a whole-body level on day 7 after treatment. We found extensive infiltration and expansion of higher affinity VHH CAR T cells (JZQ-B4, Y4A, and T2A) in the lungs, liver, and bone marrow, indicating off-tumor, normal tissue-driven proliferation (Figure 5A). Consistent with a delayed onset of systemic toxicity in mice treated with R3A CAR T cells, the expansion of R3A CAR T cells outside of tumor was notably less compared with that in other higher affinity cohorts. In comparison, specific homing to the tumor and little off-tumor expansion were seen only in the N9A cohort (Figure 5A). To our surprise, CAR T cell expansion at tumor sites was barely visible in mice treated with the higher affinity CAR T cells (JZQ-B4, Y4A, and T2A). Consistent with the imaging results, flow cytometry analysis demonstrated that T cell expansion in the lungs, indicative of systemic off-tumor expansion, was reduced with decreasing affinity of VHH CARs (Figure 5, B and C). Specifically, the frequency of T cells in the lungs of mice treated with N9A CAR T cells was similar to that of mice receiving NT cells, whereas in mice receiving higher affinity JZQ-B4, Y4A, and T2A CAR T cells, over 10% of live single cells isolated from the lungs were CD3^+^ T cells (Figure 5, B and C). IHC staining of tumor sections revealed the presence of tumor-infiltrating T cells in mice treated with lower affinity R3A and N9A CAR T cells. In contrast, minimal CD3 staining was observed in tumor samples from mice treated with higher affinity JZQ-B4, Y4A, and T2A CAR T cells (Figure 5D), corroborating the PET/CT imaging results (Figure 5A). Together, these findings underscore the potential risks associated with high-affinity CARs targeting MSLN, which may cause unpredictable on-target, off-tumor toxicities and could be responsible for the adverse events observed in clinical trials. This study highlights the substantial impact of CAR affinity on the efficacy of CAR T cells and their ability to traffic to tumor sites and serially lyse tumor cells.

## Discussion

The success of CAR T cell therapy in treating hematological malignancies has encouraged the investigation of its application in solid tumors. However, a key barrier to developing CAR T cells for patients with solid tumors is that most candidate target antigens are often coexpressed on nonmalignant tissues, leading to substantial risk of on-target, off-tumor toxicity. This toxicity, caused by CAR T cells attacking nonmalignant tissues expressing the target antigen, has been observed in various clinical trials involving patients with solid tumors (11, 19–21), highlighting the importance of establishing strategies to predict and mitigate this effect. Our group has established a robust affinity-tuning approach, through mutagenesis of the native ligand, scFv, or VHH of the CAR, to achieve an affinity sufficient for tumor cell recognition while sparing healthy cells with limited antigen expression (13, 14). In this study, we describe the affinity tuning of a VHH-based CAR against MSLN, an attractive target for CAR T cell therapy. Using a VHH that binds both human and mouse MSLN, we demonstrate the occurrence of fatal off-tumor toxicity of high-affinity MSLN CAR T cells in a mouse model. Additionally, we show that low-affinity CAR variants, produced by alanine mutagenesis of the CDRs, exhibit increased selectivity, improved tumor control, and reduced off-tumor toxicity.

The clinically severe off-tumor toxicity of MSLN-targeting CAR T cells has prompted the development of various strategies to address this safety concern. Since systemically infused CAR T cells are often sequestered in the lungs before reaching the tumor, local intrapleural delivery of MSLN CAR T cells has been attempted in patients with malignant pleural diseases (22). This approach has shown no dose-limiting toxicities or grade 5 adverse events (22). Multiple infusions of mRNA-based CAR T cells have also been well tolerated, but the transient expression of the CAR, typically lasting only a few days, may limit the long-term efficacy (23, 24). Another strategy includes incorporating a safety switch gene to allow for the elimination of CAR T cells in case of emerging adverse events, though this method may have limited effect because of incomplete elimination of the CAR T cells or excessive activation of other immune cells such as monocytes eliciting cytokine release syndrome (25–27). Our approach of tuning the affinity of the target binders focuses on discriminating differential expression of the target antigens in tumor versus normal tissues. Single–amino acid substitutions with alanine in the CDRs was able to produce anti-MSLN VHH variants with a wide range of affinities (2- to 3-log difference). High-affinity CAR T cells permitted the reactivity against target cells with a low density of MSLN expression, whereas the N9A VHH CAR variant with above 100 nM binding affinity demonstrated great discrimination between target cells with high, intermediate, and low MSLN expression levels. These findings are consistent with our previous work on generating affinity-tuned CAR T cells targeting epithelial cell adhesion molecule and intercellular adhesion molecule 1 using the same or similar approaches (13, 14).

Current FDA guidelines recommend using relevant animal models capable of eliciting a biological response that could reasonably predict the human response for preclinical assessment of investigational cellular therapy products (28). Mouse models often fail to meet this standard due to a lack of cross-species reactivity to mouse target antigens. Attempts to improve the predictiveness of mouse models include stable expression of human homologs through genetic engineering (29) and implantation of cell lines with low-density antigen expression to mimic normal tissues (30, 31). However, these approaches are rarely used and may still not accurately reflect the complex expression patterns of tumor-associated antigens on normal human tissues. Our approach, using a VHH that cross-reacts with mouse orthologs combined with CAR T cell imaging techniques, offers a distinct advantage for monitoring CAR T cell migration and on-target, off-tumor interactions in mouse models. In our preclinical study, human T cells expressing high-affinity VHH-based CARs (JZQ-B4, T2A, Y4A, and R3A) that cross-react with mouse MSLN exhibited profound off-tumor interaction and expansion. Mouse MSLN shares not only sequence similarity with human MSLN but also biochemical characteristics, tumor expression, and tissue distribution patterns (32), suggesting that the efficacy and off-tumor toxicity observed in mouse models should reflect to some extent the toxicities observed in human studies. As expected, high-affinity scFv-based AMAT CAR T cells that do not bind mouse MSLN did not induce severe toxicity in preclinical animal models. This highlights the limitations of preclinical animal studies in capturing potential off-tumor adverse events in humans for typical scFv-based human-specific CARs (33).

One limitation of the current study is that we only performed alanine substitution for the residues in the CDRs of VHH. Although we successfully identified a new low-affinity CAR construct (N9A) that reduced on-target, off-tumor effects and enhanced tumor penetration and response, a comprehensive screening of mutagenesis with other amino acids may yield variants with more ideal affinity and antitumor functionality (14, 34). For VHHs and scFvs with very high affinities, multiple iterations of mutagenesis may be required to generate variants within the optimal window of affinity. Low-affinity CAR T cells have been shown to experience reduced trogocytosis, preventing rapid antigen loss (35). On the other hand, low-affinity CAR T cells might have lower cytotoxic potency against some tumor targets and be more susceptible to tumor resistance or relapse due to antigen downregulation or loss, which is a particular challenge in solid tumors that manifest greater antigen heterogeneity (36, 37). Additional engineering strategies might be considered to potentially augment CAR T cell activity without compromising selectivity, which include coexpressing transcriptional factors like c-Jun and FOXO1 (38, 39) or locally delivering inflammatory cytokines such as IL-12 (14).

In summary, we demonstrated that systematic screening of VHHs with single–amino acid alanine substitution in the CDRs is an efficient approach to affinity tuning. Our preclinical studies showed the occurrence of fatal on-target, off-tumor toxicity by high-affinity MSLN-specific VHH CAR T cells and that optimizing the affinity of CARs can mitigate toxicity while improving tumor-targeting efficiency and safety. High-affinity CAR T cells, when sequestered in the lungs or trapped within normal organs, may even face difficulties with tumor antigen recognition and infiltration, compromising their therapeutic potential. Designing CARs with cross-species reactivity with both human and mouse antigens can improve preclinical assessment of CAR T cell products by providing more accurate prediction of tumor response and potential toxicity in patients.

## Methods

### Sex as a biological variable.

In the animal study investigating CAR T cell activity in vivo, only male mice were included in this study to minimize variability because of body weight and other sex-associated differences. Given the large number of animals required to compare 6 CAR variants and controls, we opted to use only male mice to obtain more homogeneous responses or readouts within each cohort and to avoid adjusting treatment or tumor cell doses due to the body weight differences between males and females. The relevance of these findings to female mice remains to be evaluated.

### Cell lines.

The NCI-H226 lung cancer cell line was acquired from the American Type Culture Collection (ATCC) and cultured in RPMI-1640 medium (Corning) supplemented with 10% fetal bovine serum (FBS, Gemini Bio). NCI-H226/KO cells with human MSLN knockout were generated using the Alt-R CRISPR-Cas9 System (Integrated DNA Technologies, Inc.) according to the manufacturer’s instructions. The MSTO-211H mesothelioma cell line and Jurkat T cell line were also purchased from ATCC and maintained in RPMI-1640 medium supplemented with 10% FBS. MSTO-211H cells were transduced with a lentivirus to overexpress human MSLN, resulting in the engineered MSTO-211H/hMSLN cell line. HEK293T cells were obtained from ATCC and cultured in DMEM (Corning) supplemented with 10% FBS. All tumor cells were transduced with a Firefly Luciferase-F2A-GFP lentivirus (Biosettia) for bioluminescence-based cytotoxicity and mouse imaging experiments. All cells were cultured in a humidified incubator at 37°C with 5% CO_2_ and were routinely tested for mycoplasma using a MycoAlert detection kit (Lonza).

### Discovery and characterization of VHHs binding MSLN.

Two alpacas (obtained from local alpaca farms by Tufts University) were immunized with 5 subcutaneous injections of the C-terminal domains of both recombinant human MSLN (aa 296–580, R&D Systems, Bio-Techne, catalog 3265-MS) and mouse MSLN (aa 298–600, R&D Systems, Bio-Techne, catalog 8604-MS). The first immunization dose was 150 μg of each protein, followed by 4 doses of 75 μg protein each. The final serum limit titers were approximately 1:500,000 for both immunogens in both alpacas. A nanobody-display phage library was produced from the peripheral lymphocytes collected 5 days after the fifth immunization by methods described previously (15), with a complexity of 1.2 × 10^7^. The library was panned on MaxiSorp tubes coated with 0.5 μg/mL human MSLN. Ninety-five colonies were randomly selected and screened by a modified ELISA method (15) using 96-well MaxiSorp plates (Invitrogen) coated with human and mouse MSLN, respectively. Ten clonal families were identified to bind strongly to human MSLN, and one of those displayed cross-specificity to both human and mouse MSLN. One member from each of the 10 clonal families was expressed, purified, and characterized for affinities to human and mouse MSLN by quantitative dilution ELISA (15).

### Yeast surface display and alanine scanning.

The nucleic acid sequences of the parental JZQ-B4 and its VHH variants, each with a single–amino acid substitution with alanine in the CDR1, CDR2, and CDR3, were chemically synthesized (New England Biolabs Inc.) and cloned via homologous recombination into the pNL6 vector that had been linearized with NheI and BamHI. The resulting constructs were individually transformed into EBY100 yeast cells (40) and cultured at 30°C in SDCAA medium containing 20.0 g/L dextrose, 6.7 g/L yeast nitrogen base, 5.0 g/L casamino acids, 10.19 g/L Na_2_HPO_4_•7H_2_O, and 8.56 g/L NaH_2_PO_4_•H_2_O. Surface protein expression was then induced in SGCAA medium containing D-(+)-galactose at 20°C for 24 hours. The yeast cultures were subsequently examined for binding to fluorescently labeled human MSLN by flow cytometry.

### Lentiviral vector construction.

The CAR constructs were synthesized and cloned by GenScript into a lentiviral plasmid backbone (VectorBuilder Inc., Vector Design Studio) (41) under the regulation of a human elongation factor 1α promoter. The JZQ-B4 VHH CAR construct and its affinity variants contain from the 5′-LTR end: a cMyc tag, VHH, the CD8 hinge, transmembrane and cytoplasmic domains of CD28, and the cytoplasmic domain of CD3ζ molecule. The PET reporter gene SSTR2 was incorporated at the C-terminus, following a “self-cleaving” ribosome-skipping porcine teschovirus-1 2 A sequence. The AMAT CAR was generated by replacing the VHH with scFv derived from the human MSLN antibody amatuximab.

### CAR T cell manufacturing.

Lentivirus was produced via transient transfection of HEK293T cells with a transfer plasmid and LV-MAX lentiviral packaging mix (Gibco, catalog A43237) using Lipofectamine 3000 transfection reagent (Invitrogen, catalog L3000015) following the manufacturer’s instructions. Lentiviral supernatant was collected after 72 hours, filtered through a 0.45 μm filter, and concentrated using PEG-it virus precipitation solution (System Biosciences, catalog LV825A-1). The lentivirus was aliquoted and stored at −80°C. Primary T cells were enriched from commercially obtained Leukopaks from healthy donors (AllCells) via MACS separation using CD4 (Miltenyi Biotec, catalog 130-045-101) and CD8 (Miltenyi Biotec, catalog 130-045-201) microbeads. CD4/CD8-enriched T cells were activated on day 0 with human T-Expander CD3/CD28 Dynabeads (Gibco) at a bead-to-cell ratio of 1:1 and cultured in complete T cell growth medium consisting of TexMACS medium (Miltenyi Biotec) supplemented with 5% human AB serum (MilliporeSigma), 12.5 ng/mL human IL-7 (Miltenyi Biotec), and 12.5 ng/mL human IL-15 (Miltenyi Biotec). Lentivirus transduction was performed on day 1 and day 2 after activation, followed by expansion of CAR T cells in G-Rex 6M well plate (Wilson Wolf Manufacturing). On day 10, the CAR T cell products were harvested, and CAR expression levels were determined by flow cytometry (details described in the section below). CAR T cells were normalized to contain the same percentage of CAR-expressing cells (50% CAR^+^) using donor-matched NT cells and cryopreserved in a 1:2 mixture of T cell complete growth medium and CryoStor CS10 (STEMCELL Technologies, catalog 07930). For Jurkat CAR T cells, a single lentivirus transduction was performed.

### Flow cytometry.

Cells were washed with staining buffer (PBS + 1% BSA) and stained with fluorophore-conjugated antibodies or MSLN protein at 4°C for 20 minutes. Subsequently, cells were washed twice with buffer and analyzed on a Gallios (Beckman Coulter Life Sciences) or a Symphony A5SE (BD Biosciences) flow cytometer. Data were analyzed using the FlowJo software (BD Biosciences). The antibodies used in this study include anti-human MSLN (BioLegend, clone MN, catalog 530101), APC anti-mouse IgG (BioLegend, clone Poly4053, catalog 405308), FITC anti-cMyc (Miltenyi Biotec, clone SH1-26E7.1.3, catalog 130-116-485), PE anti-human CD3 (BioLegend, clone HIT3a, catalog 300308), and PE anti-HA tag (BioLegend, clone 16B12, catalog 901518). The relative binding affinities of JZQ-B4 VHH variants displayed on the yeast surface were determined by staining cells with 300 nM of monomeric human MSLN protein (R&D Systems, Bio-Techne, catalog 3265-MS-050), conjugated in-house with Alexa Fluor 647 using a microscale labeling kit (Thermo Fisher Scientific, catalog A30009). For the saturation binding assay, Jurkat T cells expressing CAR variants were stained with 2-fold serially diluted Alexa Fluor 647–conjugated human and mouse MSLN (R&D Systems, Bio-Techne, catalog 8604-MS-050), respectively. The MFIs were used to calculate the *K_D_* using the 1-site nonlinear regression model (GraphPad Prism 10). For the experiment assessing T cell infiltration into the lungs, single cells were generated by digesting small pieces (2 to 4 mm) of lung tissue in RPMI-1640 medium supplemented with 10% FBS and 200 U/mL collagenase type IV (Gibco) for 2 hours at 37°C, followed by filtration through a 70 μm cell strainer.

### Cytotoxicity assays.

Firefly luciferase–expressing tumor target cells (NCI-H226, NCI-H226/KO, MSTO-211H, and MSTO-211H/hMSLN) were seeded in tissue culture–treated, flat-bottom, black, 96-well plates (Corning, catalog 3603) at a density of 5 × 10^3^ cells per well. Freshly thawed CAR T cells were washed and cocultured with target cells at an effector-to-target ratio of 2.5:1 in media containing 150 μg/mL d-luciferin (Gold Biotechnology). Luminescence was quantified by a microplate reader (TECAN Infinite M1000PRO) at specified time points. The percentage of viability was calculated by dividing the relative light units (RLU) by those of target cell–only wells. The cytotoxicity (%) was calculated as ([target cell–only RLU – test RLU]/target cell–only RLU) × 100%.

### In vivo mouse studies.

Male 6- to 8-week-old male NOD.Cg-Prkdc^scid^ Il2rg^tm1Wjl^/SzJ (stock no. 005557) mice were purchased from The Jackson Laboratory and housed under specific pathogen–free conditions at the Animal Core Facility of Weill Cornell Medicine. To assess the in vivo antitumor activity of MSLN CAR T cells, each mouse was subcutaneously engrafted in the upper flank with 1 × 10^6^ firefly luciferase–expressing MSTO-211H/hMSLN cells suspended in 50 μL of RPMI-1640 medium mixed with 50 μL of Matrigel (Corning). After 7 days, freshly thawed CAR T and NT control T cells were adoptively administered via tail vein injection at a dose of 1 × 10^7^ cells/mouse. Tumor burden was monitored by tumor size measurement with a caliper and bioluminescence imaging using an IVIS Spectrum imaging instrument (PerkinElmer), 15 minutes after intraperitoneal injection of 200 μL of 15 mg/mL d-luciferin (Gold Biotechnology). Tumor volume (*V*) was calculated using the formula *V* = (length × width^2^)/2. Mice were randomized to ensure uniform tumor establishment before T cell treatment. PET/CT imaging was performed to track CAR T cell biodistribution using a micro-PET/CT scanner (Inveon, Siemens) 2 hours after intravenous injection of 200 μCi ^18^F-NOTA-Octreotide tracer. PET/CT images were analyzed using AMIDE v.1.0.5. Peripheral blood samples were collected via lateral saphenous vein at indicated time points, and serum was isolated after centrifugation at 2,000*g* for 10 minutes at 4°C and stored at −80°C for cytokine analysis. Lungs and tumor samples were harvested at indicated time points for flow cytometric and IHC analyses. Mice were monitored daily by the husbandry staff and euthanized using a CO_2_ chamber upon manifestation of hunched posture, rough coat, impaired mobility, paralysis, or tumors exceeding 1,000 mm^3^.

### Cytokine analysis.

Clarified mouse plasma was quantified for cytokine levels using a LEGENDplex Human CD8/NK Panel (BioLegend) per manufacturer’s instructions. Mouse plasma from untreated mice was used as control. Cytokine concentrations were calculated using a standard curve (standards provided within the kit).

### IHC.

IHC staining was performed using the following antibodies: rabbit anti-CD3 (Ventana Medical Systems, clone 2GV6, catalog 790-4341) and HRP horse anti-rabbit IgG (Vector Laboratories, catalog MP-7401). Tumor tissues were fixed in 10% neutral buffered formalin (StatLab Medical Products), followed by embedding, sectioning, and staining according to the manufacturer’s protocol. Stained slides were imaged using an EVOS M5000 microscope.

### Statistics.

Statistical analysis was performed using Prism 10 (GraphPad). One-way and 2-way ANOVA with multiple-comparison tests were employed to evaluate statistical significance among the groups. Mouse survival curves were generated using the method of Kaplan-Meier, and significance was assessed with the log-rank (Mantel-Cox) test. *P* < 0.05 was considered statistically significant.

### Study approval.

All animal procedures were approved by the Institutional Animal Care and Use Committee at Weill Cornell Medicine.

### Data availability.

All relevant data supporting the findings of this study are reported within the article or its supplemental material, including the Supporting Data Values file.

## Author contributions

YY and MMJ designed the experiments, analyzed data, and wrote the manuscript. YY and YV manufactured CAR T cells for this study and performed the in vitro and ex vivo experiments. YA, SJT, and DSB performed animal and ex vivo experiments. JMT and CBS performed the alpaca immunization and screened for MSLN-specific VHHs. IMM discussed the results and provided valuable advice for the project. All authors reviewed and approved the manuscript.

## Supplementary Material

Supplemental data

Supporting data values

## Version 1 11/22/2024

Electronic publication
