# Supplementary material for: Affinity-tuned mesothelin CAR T cells demonstrate enhanced targeting specificity and reduced off-tumor toxicity
Source: JCI Insight. 2024 Nov 22;9(22):e186268. doi: 10.1172/jci.insight.186268 (PMC11601908; doi:10.1172/jci.insight.186268)
Supplement: Supplemental data [file jciinsight-9-186268-s032.pdf]

Supplementary Table 1. Relative binding affinities of VHH clones determined by quantitative dilution ELISA.

VHH name	EC_{50} (nM) human MSLN	EC_{50} (nM) mouse MSLN
JZQ-B2	0.5	No binding
JZQ-B3	0.2	No binding
JZQ-B4	0.2	0.2
JZQ-B7	0.2	>25
JZQ-B9	0.8	No binding
JZQ-C2	0.3	No binding
JZQ-C6	0.4	No binding
JZQ-D3	0.5	No binding
JZQ-F3	0.8	No binding
JZQ-G7	0.7	No binding
